# Supplementary material for: Extended-Spectrum β-Lactam Resistant Klebsiella pneumoniae and Escherichia coli in Wild European Hedgehogs (Erinaceus europeus) Living in Populated Areas
Source: Animals (Basel). 2021 Sep 28;11(10):2837. doi: 10.3390/ani11102837 (PMC8532684; doi:10.3390/ani11102837)
Supplement: Supplementary file 1 [file animals-11-02837-s001.zip › animals-1392879-supplementary.pdf]

Table S1. **Oligonucleotides used for the detection of ESBL/AmpC and colistin-resistance genes in this study**.

Genes		Primer sequence (5'-3)	Product size (bp)	References
bla_{TEM}	F	GCGGAACCCCTATTTG	963	Olesen *et al.,* 2004
	R	TCTAAAGTATATATGAGTAAACTTGGTCTGAC		
bla_{CTX-M}	F	ATGTGCAGYACCAGTAARGTKATGGC	593	Miro *et al.,* 2002
	R	TGGGTRAARTARGTSACCAGAAYCAGCGG		
bla_{SHV}	F	TTATCTCCCTGTTAGCCACC	797	Arlet *et al.,* 1997
	R	GATTTGCTGATTTCGCTCGG		
bla_{CMY-1}	F	ATGCAACAACGACAATCC	1085	Kim *et al.,* 1998
	R	TTGGCCAGCATGACGATG		
bla_{CMY-2}	F	GCACTTAGCCACCTATACGGCAG	758	Hasman *et al.,* 2005
	R	GCTTTTCAAGAATGCGCCAGG		
bla_{OXA}	F	GCGTGGTTAAGGATGAACAG	438	Poirel *et al.,* 2011
	R	CATCAAGTTCAACCCAACCG		
mcr-1	F	CGGTCAGTCCGTTTGTTC	309	Rebelo *et al.,* 2018
	R	CTTGGTCGGTCTGTAGGG		
mcr-2	F	TGTTGCTTGTGCCGATTGGA	567	Rebelo *et al.,* 2018
	R	AGATGGTATTGTTGGTTGCTG		
mcr-3	F	TTGGCACTGTATTTTGCATTT	542	Rebelo *et al.,* 2018
	R	TTAACGAAATTGGCTGGAACA		
mcr-4	F	ATTGGGATAGTCGCCTTTTT	487	Rebelo *et al.,* 2018
	R	TTACAGCCAGAATCATTATCA		
mcr-5	F	ATGCGGTTGTCTGCATTTATC	1644	Rebelo *et al.,* 2018
	R	TCATTGTGGTTGTCCTTTTCTG		

F, sense primer; R, antisense primer; bp, base pairs

Table S2. Odd-ratios and p-values of bivariate generalized linear models

	OR	OR CI 95%	p-value
**Age group (Model 1)**			
Adult	-	-	-
Juvenile	1.5	(0.7, 3.6)	0.249
Unknown	1.6	(0.3, 8.2)	0.549
**Sex (Model 2)**			
Female	-	-	-
Male	0.6	(0.3, 1.3)	0.192
Unknown	0.3	(0.02, 2.4)	0.326
**Month (Model 3)**			
October	-	-	-
February	0.0000001	(0, 1.4 x 10^71)	0.991
March	0.272	(0.04, 1.4)	0.145
April	0.6	(0.2, 2.5)	0.494
May	3	(0.5, 2.6)	0.262
June	0.9	(0.2, 3.5)	0.878
July	1.2	(0.2, 6)	0.822
August	1.1	(0.2, 6.5)	0.895
September	2.3	(0.3, 2)	0.427
November	1.3	(0.3, 5.4)	0.727
December	0.6	(0.07, 3.6)	0.592
**Cause of admission (Model 4)**			
Fortuitous	-	-	-
Accident	1.2	(0.3, 6.1)	0.8275
Breeding	1.6	(0.5, 4.9)	0.404
Trauma	1.1	(0.3, 4.3)	0.878
Weakness	0.8	(0.2, 2.7)	0.724
Other	1.4	(0.2, 5)	0.599
**Population density (Model 5)**			
>1000 ha/m2	-	-	-
1000-2250 ha/m2	2.1	(0.7, 6.5)	0.202
2250-5000 ha/m2	1.2	(0.4, 3.8)	0.769
>5000 ha/m2	1.3	(0.4, 4.2)	0.631

Table S3. Odd-ratios and p-values of multivariate generalized linear models

	OR	OR CI 95 %	p-value
**Age group**			
Adult	-		-
Juvenile	1.375	(0.5, 3.6)	0.515
Unknown	2.235	(0.2, 2.4)	0.497
**Sex**			
Female	-		-
Male	0.586	(0.2, 1.6)	0.291
Unknown	0.00000003	(0, 2.8 x 10^118)	0.994
**Month**			
October	-		-
February	0.00000003	(0, 3.9 x 10^118)	0.994
March	0.276	(0.03, 1.8)	0.196
April	0.338	(0.1, 1.9)	0.228
May	2.476	(0.3, 27.1)	0.424
June	0.723	(0.2, 3.2)	0.664
July	1.041	(0.2, 5.7)	0.962
August	1.492	(0.2, 11,9)	0.696
September	1.679	(0.2, 16.9)	0.634
November	0.969	(0.2, 4.6)	0.969
December	0.236	(0.01, 2)	0.24
**Population density**			
>1000 ha/m2	-	-	-
1000-2250 ha/m2	3.073	(0.9, 11.6)	0.084
2250-5000 ha/m2	1.426	(0.4, 5.5)	0.5976
>5000 ha/m2	1.994	(0.5, 8.4)	0.332
